# Building effective service delivery mechanisms for justice-involved individuals: an under-researched area

**DOI:** 10.1186/2194-7899-2-2

**Published:** 2014-01-29

**Authors:** Faye S Taxman

**Affiliations:** Department of Criminology, Law and Society, George Mason University, 4087 University Blvd, Ste 4100, Fairfax, VA 20030 USA

## Abstract

**Background:**

The *Heath & Justice* journal is devoted to addressing the unmet needs of those involved in or working in the justice system. With attention to both health and justice processes and outcomes, this journal is designed to provide a forum for scholarship and research that is usually dispersed across many different disciplines.

**Findings:**

In this article, we focus on the need for more service related research to broaden our understanding of how to improve system, program, and client level outcomes. A review of pertinent research in each area is provided to illustrate contemporary findings.

**Conclusions:**

Current research also makes the case for a focused discussion about processes, policies, and procedures that need further exploration. To better understand how to improve health and justice outcomes, research is needed in program fidelity, services, geographical and activity spaces, and other arenas that affect individual, program, and system level outcomes.

**Electronic supplementary material:**

The online version of this article (doi:10.1186/2194-7899-2-2) contains supplementary material, which is available to authorized users.

## Background

The high rate of infectious diseases, behavioral health disorders, and some chronic diseases among justice-involved offenders alone makes the case for a specialized journal devoted to *Health & Justice*. Offenders in prison and jail have more access to health services than when they are in the community and few offenders in the community have ready access to behavioral health or infectious disease services (Cropsey, et al. 2012). Given the many co-morbid conditions, the necessity for a specialized journal is even more pronounced given the challenges of contemporary justice and health care delivery systems in addressing these complex issues that affect both health and safety outcomes. The promise for this journal, as outlined in the journal aims (http://www.healthandjusticejournal.com/about) is to expand and enlarge the most frequently asked question—what type of programming or health care produces better outcomes. The scientific questions are geared toward the need for better, and perhaps different policies, practices, and procedures that affect health and justice related desired outcome(s).

A range scientific methods are more adept at studying a broader array of questions that examine the processes and system of care (health services, process studies), how the various system affects outcomes (health services), the degree to which the policies and/or practices are implemented with fidelity (implementation science), the best way to practice more research-based findings (translational science), and the optimum way to address the needs of stakeholders (community participatory research frameworks). While traditional efficacy trials answer one set of questions, efficicay studies do not address broader questions about who delivers care, how care is delivered, in what settings, under what conditions or optimal conditions, and how to improve care. The list of unanswered questions that pertain to how services are delivered and the impact of policies, practices and procedures on health outcomes are front and center in the advancement of scientific findings into practice. These scientific venues challenge us to examine how different factors--whether it is the characteristics of an individual, a program, the organization, set of processes, or systems-- affect the processes by which justice and health care are utilized and how improvements in individual level (client) and system outcomes can occur. *Health & Justice* intends to cover this gamut of related questions and issues directed towards efforts to advance practice and inform these practices through research and science. Our attention draws to the broader array of issues that affect individual, program, and organizational level outcomes.

Over the last decade there is emerging research on the challenges, barriers, and facilitators of client-level outcomes. This research points to the organization and delivery systems, and how they affect the decisions made by individuals, either clients of the justice and/or health systems or workers in these delivery systems. This essay briefly summarizes interdisciplinary knowledge basis about service process and delivery issues consisting of various types of scientific endeavors that are pertinent to the issues relevant to this *Health & Justice* journal. This article outlines the current knowledge basis about program fidelity and components that drive better quality programming, impact of service delivery processes that affect outcomes, impact of geography and spatial issues related to service delivery, and impact of individual level factors and health and justice disparities that affect programming. In an era with rapidly changing justice and health care systems in the U.S. and elsewhere around the world, quality research can contribute to marked improvements in our knowledge about efficacious, cost-effective, and racially and class neutral efforts to improve public health and justice related outcomes. The goal of the *Health & Justice* journal is to be a targeted outlet for this complex array of research, along with opportunities to contribute to the field with improved research protocols, implementation studies, training tools, and other research-based products that can assist with the adoption, implementation, and sustainability of desired practices.

## Program fidelity and components

While program design and fidelity are intertwined, a good design may not be enough to guarantee good program outcomes. The delivery of key program components that facilitate change within an individual is needed to achieve these outcomes. A major theme present in the evidence-based practices literature is that the practices need to be of high integrity, both design and delivery, to achieve the desired outcomes. Fidelity generally refers to adherence to a particular treatment approach and orientation, including the use of a manual, using the prescribed dosage and frequency of sessions, and delivering similar to the research-basis. Quality practices and policies yield better program outcomes, which generate improved client-level outcomes in at least one area such as addiction treatment, correctional programming, and HIV care. This design-implementation-fidelity paradox requires an appreciation for the factors that are needed to produce positive outcomes. Much of the evidence-based practices literature emphasizes structural issues—such as what assessment tools to use, who is the eligible population, what therapies or curriculums to use, for how long, who should deliver, and so on. These are all critical questions but they do not address the barriers or facilitators that allow the design to be actualized. As noted by implementation scientist expert Dean Fixsen, “…implementation frameworks are not an end point, but a new beginning for expansion of knowledge related to implementation best practices, science, and policy” (http://nirn.fpg.unc.edu/about-nirn/our-approach).

Understanding and measuring program quality is now part of the cadre’ of endeavors to drive and facilitate better outcomes. In the mental health field, McGrew et al. (1994) report that programs that scored higher on program integrity (i.e. fidelity to a specified program model) yield greater impact on addressing client symptoms. The measure of program integrity included: 1) program components (and clinical orientation), 2) program dosage or the number of treatment hours and sessions over a period of time, 3) staff certification and training, 4) program management, quality assurance, and quality controls, 5) presence and use of an evaluator, and 6) program benchmarks and quality control. These are the consistent themes across the disciplines.

In the criminal justice discipline, studies reiterate the importance of program quality to reduce negative justice outcomes of arrest and incarceration in justice-involved individuals (Lowenkamp and Latessa 2005; Lipsey 2009). Recent meta-analyses review the components of program implementation that yield better outcomes both in terms of participation in the program and/or client outcomes, and mirror the mental health field in terms of key components. In a review of 273 studies, Andrews and Dowden (2005) found larger effect sizes for programs with integrity, specifically programs that adhered to a specific treatment model, involved staff with good interpersonal skills and trained in the delivery of a specific program, included clinical supervision by an individual trained in the program, and an evaluator was involved in program design or implementation. Lipsey and Landenberger (2006) reported that program design (use of cognitive-behavioral programming), presence of an evaluator, and an increased number of treatment sessions had a positive impact on client-level outcomes. Similarly, in a meta-analysis of juvenile justice programs, Lipsey (2009) reported that larger effect sizes are obtained when program components included counseling, skill building for the clients, and interventions were built on an effective intervention such as family therapies or cognitive behavioral therapy. To that end, several different program assessment tools are available to assess quality of programming such as the *The Program Tool* in the RNR Simulation Tool (Crites and Taxman 2013; see http://www.gmuace.org/tools), the SPEP (Lipsey et al. 2007), and the Correctional Program Assessment Inventory (Gendreau and Andrews 1994).

Another approach to defining and assessing quality is to define a set of principles as a foundation to build evidence-based practices on, and then measure the degree to which these principles are put into place. The National Institute on Drug Abuse’s *Principles of Drug Addiction Treatment: A Research-Based Guide* (National Institute on Drug Abuse 1999; http://www.drugabuse.gov/PODAT/) and its companion version for criminal justice-involved offenders (Fletcher and Chandler 2006) are examples of a set of principles. The principles articulate both effective program practices and service delivery issues, and provide ready access to the research literature on “what works”. The 13 principles are: 1) Addiction is a brain disease and no single treatment is appropriate; 2) Treatment needs to be readily available; 3) Effective treatment attends to multiple needs of the individual, not just drug use; 4) Treatment needs to be flexible; 5) Remaining in treatment for an adequate period of time is critical for treatment effectiveness; 6) Individual and/or group counseling and other behavioral therapies are critical components of effective treatment for addiction; 7) Medications are an important element of treatment for many patients; 8) Addicted or drug-abusing individuals with coexisting mental disorders should have both disorders treated in an integrated way; 9) Medical detoxification is only the first stage of addiction treatment; 10) Treatment does not need to be voluntary to be effective; 11) Possible drug use during treatment must be monitored continuously; 12) Treatment programs should provide assessment for HIV/AIDS, Hepatitis B and C, tuberculosis and other infectious diseases; and 13) Recovery from drug addiction can be a long-term process. These principles refer to the system of care (principles 1, 2, 10, 12, and 13) and direct clinical programming (3, 4, 5,6, 7, 8, 9). The version for criminal justice-involved populations includes the use of sanctions and rewards. NIDA researchers designed these principles to articulate a measure of fidelity for systems and programs to use to be “research based”.

Pearson et al. (2012) conducted a meta-analyses of 232 studies that examined the original 13 NIDA principles to examine how the principles affect client-level outcomes (generally drug use). Some of the principles were not included in the meta-analyses because they did not lend themselves to ethically –based clinical trials such as drug abuse is a brain disease (1), the earlier treatment is offered in the disease process (3), medically assisted detox is the first stage of treatment (10), use of medications (7), and attention to multiple behavioral health issues (9) and use of coercion (11). Of the remaining seven principles, research of the impact of these principles includes: matching treatment to client needs (2) (range of effect size of .11 to .38), attending to multiple needs (4) (.01 to .04), remaining in treatment (5) (.00 to .03, ns), treatment plan reassessment (8) (.05 to .46), drug testing during treatment (12) (-.05-.13, ns), and counseling to reduce HIV (13) (.002 to .038). The meta-analysis found differential effect sizes for different strategies including contingency management (.13-.29), cognitive behavioral therapy (.02 to .20), and therapeutic community (.18 to .53). The range of effective sizes was due to the quality of the study (with lower quality studies having larger effect sizes) and publication bias (published studies had larger effect sizes). From a quality perspective, these principles and their reported effect size provide guidance as to the type of factors that are important in program quality.

In the justice literature, a number of scholars and practitioners have articulated a similar set of evidence-based practices principles for correctional programming. One frequently cited set of principles is the National Institute of Corrections’ principles of effective correctional management of offenders in the community (National Institute of Corrections 2004) which echoes several of the same principles articulated in the NIDA 13 principles. The NIC model expands the principles to include organizational development (to address implementation and sustainability issues) and collaborations (to garner stakeholder support for the delivery of evidence-based practices across organizational and agency efforts including community treatment providers). The core elements are: 1) assess actuarial criminal justice risk and dynamic needs; 2) enhance intrinsic motivation; 3) target assignment of offenders to interventions based on risk level, need level, dosage of program, and integrate into full sentence; 4) provide staff with skills to deliver the evidence-based practices; 5) use rewards to improve outcomes; 6) engage support from the community; 7) measure relevant process and practices; and 8) provide feedback. Principles 4, 6, 7 and 8 refer to system and organizational issues whereas principles 1, 2, 3, and 5 refer to programming issues. A meta-analysis of these principles has not been conducted yet but Prendergast and colleagues did report that adherence to the Andrews and Bonta Risk-Need-Responsivity model (partially included in the NIC principles) does appear to impact reductions in crime but not drug use (Prendergast, et al. 2013). Taxman and Viglione (2013) recently summarized the major research findings from the NIC specified evidence-based practices regarding challenges to putting the NIC principles into place and recent advancements to address these research needs, as shown in Table 1. This table also outlines some valuable arenas for advancing our knowledge about evidence-based practices in the realm of justice organizations that are moving forward with improving the quality of practices and services delivered.

**Table 1:** **Barriers and facilitators to EBP implementation**

EBP Area	Findings	Recent advancements
**Risk and needs assessment**	Officer skills and attitudes are not consistent with the use of the tools (Farrell et al. 2011)	Management and organizational support for validated risk and need assessment tool
	POs do not use assessment results to determine risk level or frequency of contact (Bonta et al. 2008; Miller and Maloney 2013)	Use of officer skill development curriculum to advance focus on problem solving (STICS curriculum (Bonta et al. 2011 2008; Bourgon et al. 2010; Bourgon and Gutierrez, 2012; EPICS curriculum (Smith et al. 2012); STARR curriculum (Robinson et al. 2011); PCS curriculum (Taxman 2002 2008); SOARING2 curriculum (Taxman, et al. 2013; Maass, 2013)
	Officers do not use the instruments and do not comply with assessment (Miller and Maloney 2013)	External facilitators (Taxman, et al. 2012)
	Lack of trust in assessment tool (Krysik and LeCroy 2002; Miller and Maloney 2013)	Coaching (Baer 2006; Young et al. 2013)
	Prefer their own judgment (Hilton and Simmons 2001) and loss of discretion (Ferguson 2002)	Address staff concerns specifically in training (Ferguson 2002)
	Many agencies do not assess offenders due to lack of time (Latessa et al. 2002)	Make sure training covers how the tools help POs to do their job more effectively (Ferguson 2002)
	Use of outdated, poorly designed and/or empirically unvalidated classification instruments (Latessa et al. 2002; Matthews et al. 2001; Latessa 2002)	Work on value clarification to advance use of tools (Miller and Maloney 2013)
**Offender engagement**	Lack of offender participation in the process and overall buy-in	Effective use of MI (McMurran 2009) and other communication tools (Taxman 2008)
	Staff training and skills in areas of engagement (Taxman 2002; McMurran 2009)	Organizational goals that reflect values associated with using behavioral management strategies (Taxman 2008)
	Authoritarian (legal) vs. shared decision-making (Taxman 2006)	Build rapport early in the supervision process between PO and offender (Taxman 2002; Robinson et al. 2011)
	Assessment and supervision process is focused on “paperwork” and not client centered (Taxman 2002)	Increasing the number of individual counseling sessions during the first month of treatment (De Leon 1991)
	Offender not ready for change (Prochaska et al. 1992; Simpson 2004)	
**Case planning and supervision plans**	Probation staff feel they do enough paperwork and the case plan is just the same as the court-ordered conditions;	Specialized curriculum work on tailoring case plans to RNA and also focusing on problem solving (STICS curriculum --Bonta et al. 2011; Bonta et al. 2008; Bourgon et al. 2010; Bourgon and Gutierrez 2012; EPICS curriculum --Smith et al. 2012; Starr curriculum --Robinson et al. 2011; PCS curriculum-- Taxman et al. 2004; Taxman 2008)
	Offender is often not involved in the case planning process; don’t use risk assessment information to inform the case plan (Taxman 2006)	Use of performance measures for supervision staff, offenders, and organizations (review case plan monthly and base review on case plan accomplishments) (Taxman 2008)
	Link between risk and need assessment and case planning is difficult (Miller and Maloney 2013)	
**Treatment programs**	Overall resistance to treatment programming since they are considered “extra”, “an opportunity” (Paparozzi and Gendreau 2005; Taxman and Bouffard 2002; Thanner and Taxman 2003)	Organizational culture that fosters performance achievement and backs it up with training and internal support for its employees will likely value and seek to implement higher quality programming, including EBPs (Friedmann et al. 2007)
	Belief that high-risk offenders will not respond to treatment (Thanner and Taxman 2003)	Administrators with a background in human services, knowledge about EBPs and a favorable attitude toward rehabilitation have the opportunity and power to set informed priorities and policies to improve services for drug-involved offenders (Friedmann et al. 2007)
	Rejection of research/Ignorance of crime and its cures (Latessa et al. 2002)	Organizations better integrated with community-based service providers (Taxman et al. 2009)
	Do not use treatment models that are proven effective (Latessa 2002; Matthews et al. 2001)	Lower levels of cynicism for change (Farrell et al. 2011)
	Interventions often do not conform to the principles of effective intervention – only in 13% of the time (Andrews et al. 1999; Friedmann et al. 2007)	Positive perceptions of leadership (Farrell et al. 2011)
	Correctional agencies traditionally do not require systematic evaluate effectiveness (Gendreau et al. 2001)	Include intent of reform in training (Steiner et al. 2011)
		Increase involvement of line-staff in change process (Steiner et al. 2011)
		Focus on fidelity to the key treatment principles
**Supervision (required conditions)**	Lack of understanding of what is quality supervision (Taxman 2012)	New mission, values, and goals (Taxman 2012)
	Organizational culture affects the professional identity which is compliance and management of risk behaviors (Durnescu 2013)	Organizational culture that fosters performance achievement and backs it up with training and internal support for its employees will likely value and seek to implement higher quality programming, including EBPs (Friedmann et al. 2007)
	Still focusing on face-to-face contacts as “check-ins” as compared to behavioral management	Administrators with a background in human services, knowledge about EBPs and a favorable attitude toward rehabilitation have the opportunity and power to set informed priorities and policies to improve services for drug-involved offenders (Friedmann et al. 2007)
	POs pay little attention to criminogenic needs (Bonta et al. 2008)	
		Organizations better integrated with community-based service providers (Taxman et al. 2009)
		Lower levels of cynicism for change (Farrell et al. 2011)
		Positive perceptions of leadership (Farrell et al. 2011)
		Include intent of reform in training (Steiner et al. 2011)
		Increase involvement of line-staff in change process (Steiner et al. 2011)
**Problem solving**	Lack of skill development by POs (Pullen 1996; Bonta, et al. et al. 2008; Taxman 2002)	Various new curriculums work on these problem solving skills
	Lack of PO buy-in (Pullen 1996)	Reduction of caseload size and attention to outcomes
	Lack of analytical framework in terms of understanding patterns and trends	
	Professional socialization and culture reinforce compliance issues (Durnescu 2013)	
**Rewards**	Punitive/control environment (Rudes et al. 2012)	Quality improvement and PDSA process to identify system issues to support the use of rewards (Rudes et al. 2012)
	Emphasis on sanctions (California Association of Drug Court Professionals 1997; Cooper 1995; Goldkamp 1994; Lindquist et al. 2006; Rudes et al. 2012; Terry 1999)—drives the environment	Management endorsement of rewards, and focusing on outcomes (Friedmann et al. 2007)
	Generally can not deliver sanctions in swift, certain pattern (Rossman, et al. 2011; Rudes, et al. et al. 2012)	Use of simple strategies (such as fishbowls) over more complex strategies (Stitzer et al. (2010)
	Generally support the use of rewards (Murphy et al. 2012) but attitudes toward rewards depends on perception of offender(Rudes et al. 2012)	
	Lack of predetermined reward schedule (Miethe et al. 2000)	
	Rewards less specific, less swiftly applied, more subjective than application of sanctions (Lindquist et al. 2006)	

Generally, the literature has evolved to the point where we have a better sense of the factors that affect program quality. Well-articulated principles are an important component since they provide guidance as to “what it is” (i.e. what the research is really saying, what are the key components, how would one know if they are “doing quality”). But to a large extent, while our knowledge has grown over the past few years, there are many areas where more research is needed to guide practices that can improve outcomes.

## Delivery processes in justice and/or health settings

Health and justice have varying outcomes of interest. In the health field, the general issue is symptom reduction or remission of a chronic disease. In the justice field, recidivism or the future involvement in the justice system (i.e. rearrest, reconviction, reincarceration, and technical violations) is of primary concern. For justice-involved individuals, health and justice outcomes are both of interest given that comorbid conditions are costly to society with more expensive behavioral and somatic health care and more involvement with the justice system. The question frequently asked, but oftentimes unanswered, is whether improvements in symptom reduction or remission of a disease will ultimately contribute to reductions in justice involvement. In the substance abuse treatment literature, the answer to the question is affirmative for heroin addicts specifically (Nurco et al. 1984; Taylor, et al. et al. 2001) and generally substance abuse treatment for those involved in the justice system (Hubbard, et al. 1997; National Institute on Drug Abuse 2012; Wooditch, Tang and Taxman 2013). In fact, a recent study of drug-involved probationers involving time-varying models found that involvement in treatment shortly after being placed on probation has a longer term impact on reductions in drug use and rearrest. What is unique about this study is that it demonstrates the value of substance abuse treatment when controlling for risk level of offender (likelihood of recidivating) and examining changes in needs (i.e. family networks, friends, employment, etc.) on positive outcomes in health and justice (Wooditch, Tang, and Taxman 2013). This study illustrates how program participation affects individual level factors in terms of changes in the individual with a constructive impact on reduced drug use and reduced justice involvement. More studies in this domain are needed.

Within the last two decades, strides in the addiction treatment and HIV/AIDS care literature have demonstrated the general importance of processes that facilitate engagement in care, and that influence overall health and/or justice outcomes. This body of research is emerging, particularly as part of implementation science methods unfolds in the greater health and justice venues. One clear advantage of this approach is the attention to variables that measure a number of processes of care that facilitate outcomes. That is, it is not always the case that “if you build it, they will come”. In fact, many programs falter because of too few participants or too few eligible participants enroll in a program.

In the addiction treatment field, a group of researchers and practitioners proffered measures to understand the process of care. In 1998, three measures were identified: identification (% of the enrollees in a health system with a diagnosis of substance abuse disorders), treatment initiation (% with admission to treatment within 14 days of identification or assessment), and engagement (% of diagnosed SUD with two treatment services in 30 days after initiation of care or assessment) (Garnick, et al. 2009). The same group formed a workgroup on public sector applications in 2004 to advance the use of these measures for clients in public programs, and to be compatible with existing data bases. The workgroup expanded existing measures to include continuity of care or continuing treatment after assessment, detoxification, short and long-term residential, or inpatient treatment (Garnick, et al. 2009). A study of the predicative validity of the measures found that clients who initiated and were engaged in care had reduced rearrest and reincarceration rates as compared to those who only initiated treatment but did not participate in care (Garnick, et al. 2007). The emphasis on process issues fosters other innovations such as quality improvements or the Network for the Improvement of Addiction Treatment (NIATx) (http://www.niatx.net) that emphasizes strategies to reduce wait lists, increase admissions to treatment, and increase retention in treatment. Researchers have found that strategies that reduce waitlists and accelerate initiation of treatment can improve retention in treatment (Johnson, et al. 2008; McCarty, et al. et al. 2007). Focusing on process has several advantages, yet there has been little attention on improvements in processes to accelerate initiation, engagement, and retention in behavioral health treatment after arrest or at any stage in the justice system (McCarty and Chandler 2009; Taxman et al. 2009). Garnick et al. (2007) illustrated how improvements in process can impact rearrests but this is just the beginning of examining service process indicators.

Research in HIV care for justice-involved individuals illustrates that there is great potential in focusing on these service-process related issues to improve outcomes. HIV positive inmates tend to have poor adherence to medications and their increased RNA levels are associated with increased spread of the infection to others (Anderson 1988; Hollingsworth et al. 2008), often due to return to high risk behaviors of unprotected sex, shared needles, and other behaviors. It is important to find strategies to promote adherence to medications, as interventions that promote HAART adherence (antiretroviral therapy) should advance the individual well-being and public health. Providing HAART during incarceration poses a significant challenge. In a retrospective analysis of 292 HIV + inmates receiving HAART from 1997 to 2002 in prison and then re-incarcerated after having spent ≥3 months outside the prison, Springer et al. (2004) found that taking medication in prison reduced the HIV RNA to 1.04 log_10_ copies/mL and those that returned to prison had elevated HIV-1 RNA level by 1.14 log_10_ copies/mL (*P* < 0.0001). This included a mean increase in the CD4 cell count of 67 cells/mm^3^ between incarceration periods. The prison sample had a decrease in the CD4 cell count of 80 cells/mm^3^ during the period of release into the community (*P* < 0.0001). Even when returning individuals were given a prescription for HAART, few filled their prescription within 10 days of release (Baillargeon et al. 2009). Favored solutions to improve HIV outcomes are case management and linkage to care, antiretroviral adherence support, treatment of substance abuse disorders, treatment of mental illness and HIV risk reduction interventions (Springer et al. 2011). Beginning methadone maintenance in prison for opioid dependent individuals appears to increase retention in substance abuse care after release and decrease drug use (Kinlock et al. 2009). Retaining patients on medication assisted treatment after release for 24 weeks increases the odds of a reduced viral load for HIV + patients (Springer et al. 2012). There are benefits to beginning treatment in prison and/or jail, but the issues related to continuity of care needs attention. Studies of pre-release versus transitional case management did not find any difference in retention in HIV care, HIV treatment outcomes, follow-up visits, emergency room visits, or reincarceration for prisoners with HIV + (Lincoln et al. 2006; Rich et al. 2001; Zaller et al. 2008).

**Figure 1:**
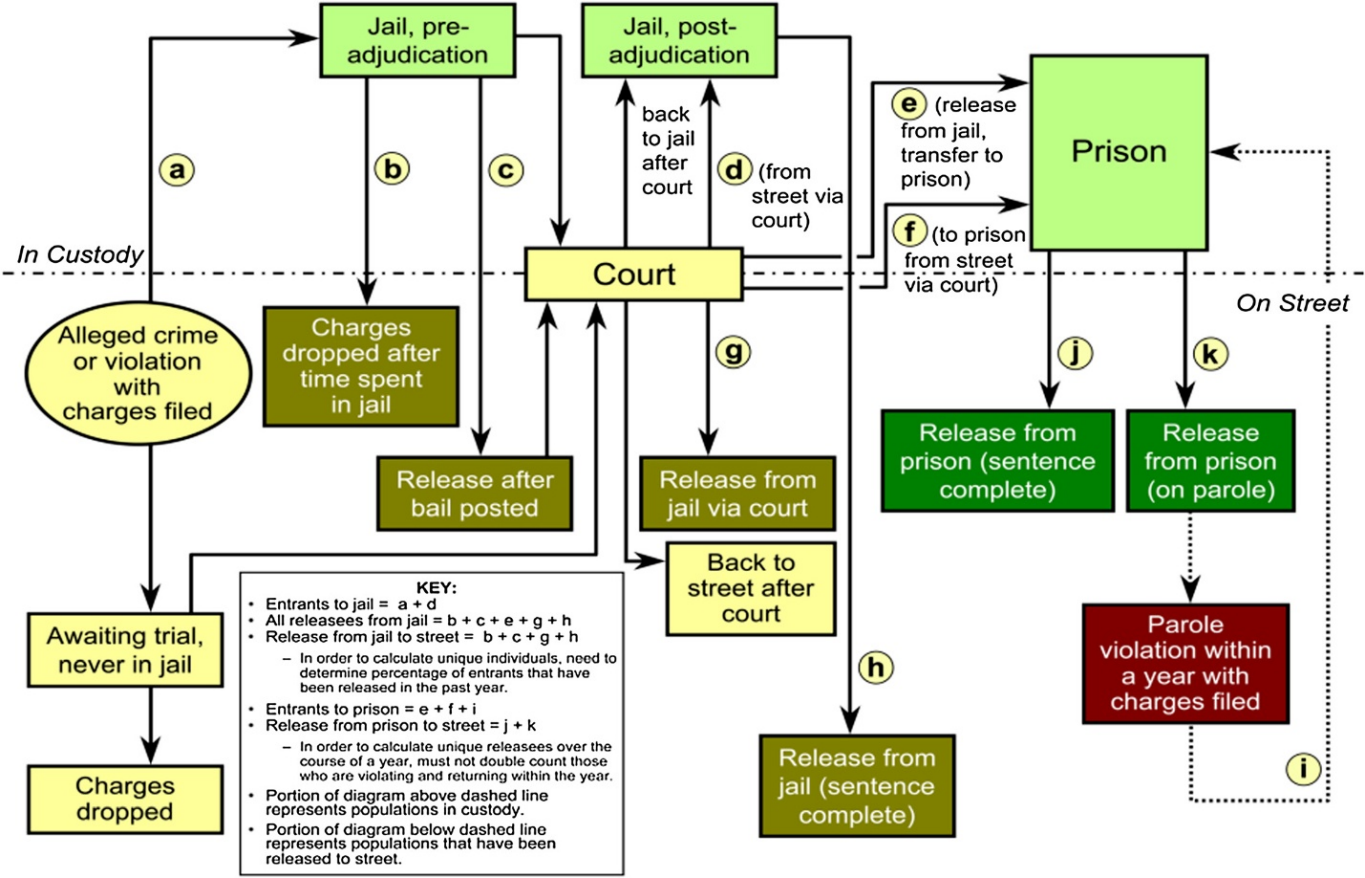
**Potential service delivery points in the justice system Spaulding, 2012.**

One critical component to prevent the spread of HIV is the identification of individuals that are HIV+. Kavasery et al. (2009) evaluated the optimal time period to test newly incarcerated jail detainees for HIV using an opt-out strategy, an approach that informs the patient that they will be tested unless the person explicitly declines. In two controlled trials of 298 males and 323 females newly incarcerated, routine opt-out HIV testing was offered at one of three points after incarceration: immediate (same day), early (within the next day), or delayed (7 days). The proportion of men and women in each group consenting to HIV testing was the outcome. Agreement to be tested was significantly higher in the early (45%) and immediate (53%) testing groups compared to the delayed (33%) testing group in males. For females, the early testing group (73%) had a significantly higher percentage of HIV testing. The study illustrated that HIV testing was feasible and acceptable by the patients within 24 hours of incarceration. This suggests that identification of HIV among justice-involved individuals is possible with quality screening and assessment processes. Questions remain regarding where and when to test for HIV, and what type of care can advance adherence to HIV care and medications. Spaulding et al. (2009) illustrates the various points in the justice process where HIV testing and care can be delivered, as shown in Figure 1. This roadmap provides many different avenues for evaluating processes and service delivery components that need exploration.

HIV researchers have taken a services research approach to examine how to increase involvement in care that can reduce infection rates amongst the general and justice-involved populations. In order to improve outcomes, researchers developed a comprehensive approach, which is referred to as Seek-Test-Treat-Retain (STTR), a model that the National Institute on Drug Abuse has over 23 grantees to test various strategies (http://www.drugabuse.gov/researchers/research-resources/data-harmonization-projects/seek-test-treat-retain). As part of this continuum of care model, a number of intertwining goals are to identify newly infected or at-risk individuals, diagnosis, link to care, retain in care, prescribe medication, and measure viral loads and suppression. Researchers use this model to estimate the impact of policies and practices at various levels, as well as identify new approaches to advance the STTR goals. The goal is to find ways to engage nearly 850,000 people who need suppression of their viral loads to reduce transmission of HIV infections. The STTR model can extend to many other unexplored behavioral health issues to identify justice-involved populations ‘specific needs, identify issues and processes related to advancing the access to services, initiate needed services, and make progress to advance more desirable longer term outcomes of reduced symptoms and improved justice outcomes.

An important service delivery issue is understanding how engagement and retention in care can reduce justice outcomes. Research in substance abuse treatment for those with high tolerance for drugs has demonstrated an impact on recidivism but this is not true for mental health, substance abuse, employment issues, and other social, behavioral health issues. The impact of quality care for somatic health on justice outcomes is an area that requires future research. The focus on processes of care involved in seeking and maintaining involvement in health and justice care for a wide range of social, behavioral, and physical issues is important. Given that health disparities and citizen disenfranchisement (i.e. loss of privileges such as voting, housing, etc.) can interfere with justice-involved individual pursuing services, a challenge is to develop processes that are both positive and address these social barriers. Much will be learned as a result of the need to enroll justice-involved individuals in the Affordable Health Care Act given that an estimated 245,000 new prison releases are likely to be eligible for insurance and another 172,000 could be eligible for tax credits to defray the cost of insurance (Cuellar and Cheema 2012). More importantly, changes in policies and practices may reduce the demands on access to ACA such as suspending Medicaid during the period of incarceration instead of terminating benefits. Such process improvements are considered useful to advance health care outcomes overall.

**Figure 2:**
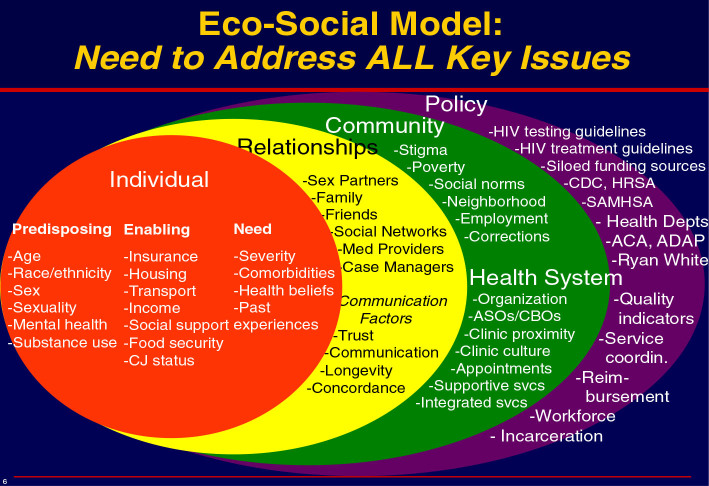
**Health-Justice Framework (Altice,** 2013 **).**

The Andersen Behavioral Model of Health Services Use (Andersen 1995 2008; Andersen and Newman 1973) identifies four primary determinants of health service use: predisposing characteristics (e.g., demographics, social structural factors, and health beliefs), enabling characteristics (e.g., individual and community resources that facilitate service use, such as financial resources and health insurance), need (e.g., a person’s general health and functional state), and health system features (e.g., environmental and contextual factors, including local health care policies). Altice (2013) added enabling factors to include criminal justice status, severity of needs (i.e. comorbidities, prior experience, etc.), and community level factors. In this revised model, Altice (2013) identified a cadre’ of policies that need further testing to determine factors that affect health service use. These policies include HIV testing and treatment guidelines, siloed funding streams, CDC/HRSA/SAMSHA/Ryan White, health disparities, quality of care indicators, service coordination, and reimbursement (Figure 2).

## Impact of geography and spatial issues on offender outcomes

Geographical studies related to access and retention in services are an emerging area that includes the role of individual and community networks in facilitating access and retention in care. The “$1 million dollar blocks” refers to neighborhoods and communities with concentrations of justice-involved individuals and an array of justice and social services expended on the population. Rose and Clear (2002) described how one such neighborhood is depleted in protective community anchors and networks that reduce disorder and contribute to healthy citizens. In assessing one Oregon community, Kubrin and Stewart (2006) reported that parolees living in areas with high concentrations of justice-involved individuals had an increased likelihood of rearrest and incarceration. Wooditch, Lawton and Taxman (2013) found that the availability of drugs (as measured by calls for service) in a probationer’s neighborhood increased drug use among probationers that have some drug-involvement. These studies illustrate how neighborhoods affect negative outcomes of individuals.

A recent study of parolees in California documented the importance of having service providers within a reasonable distance of concentrations of parolees (Hipp et al. 2010). The likelihood of recidivism is reduced by 41% when parolees reside within two miles of any service provider. African Americans living within a reasonable distance to seven service providers have the same risk of recidivating as white parolees with no service providers nearby. Service providers located in close proximity to residences of parolees increases the utilization of services (Hipp et al. 2010). A collateral finding is that service providers in communities with large concentration of parolees can become overloaded, which ultimately may have a negative impact on parolee outcomes including inability to provide adequate health care (Hipp et al. 2009).

These studies illustrate the importance of an individual’s “activity space” and the location of key geographical features within that “space” that can serve as risk and protective factors on individual level decisions. Specifically, the features of a place may affect the willingness and motivation to participate in treatment (Archibald 2008; Jacobson 2004; McLafferty 2008; Sherman et al. 2005). Each key geographical location of where an individual lives, works, receives treatment, is supervised, or engages in other mainstream activities has physical attributes that may affect psychological processes that facilitate or impede relapse or retention in treatment (Davis and Tunks 1990–1991; Jacobson 2004). Physical attributes that may have such negative effects include vibrant drug markets, which can present “frequent cues to drug taking” (Brown et al. 2004; Wooditch, Lawton, and Taxman 2013) or opportunities to become involved in the drug trade or ancillary activities (e.g., prostitution for drugs). Similarly, high levels of street violence may generate enough fear to restrict an individual’s movement and routines. Roman and Chalfin (2008) identify how violence may make people afraid to walk outside their homes. Visible signs of disorder may be an indication of a community’s inability to protect itself against crime (Skogan 1992; Yang 2007), and may be an indication of a lack of informal and formal social controls in that location. In fact, social networks and how they influence the use of space, and the impact on decisions to engage in care among justice-involved individuals is an area where more research is needed. Greater attention to how the physical environmental and place within an individual’s activity space can impact individual level decisions is warranted, as well as a better understanding of the social networks within these activity space that also influence behaviors.

## The many issues related to services and process

An important question is whether the criminal justice system is part of the service provider network in many communities. Being part of a service provider network requires the “feeder” or component to have sufficient access to a population that needs certain services and the ability to provide such services. Research documents the unmet socio-psychological and somatic needs present within justice-involved populations, including the higher prevalence of substance use disorders, mental health disorders, and infectious diseases as compared to the general population. These conditions comingle with criminal risk behaviors, creating a complex structure of needs requiring the attention of health and safety agencies. Over the last few decades, various demonstrations and studies have shown how the justice system can become part of a service provider network including referring for services in the community, offering drug treatment and problem solving courts, offering HIV testing and care, providing a myriad of programming to address various risk and need factors of the individual, and some community-based interventions. The Anderson Behavioral Model of Health Services (and modified model offered by Altice, 2013) includes justice-related issues as part of both the individual and community needs. This dual inclusion is justified by the size and scope of the justice population, and the degree to which mass incarceration policies have resulted in justice involvement as being a distinctive, but common, factor. Treating criminal offending as a public health factor advances the notion that offending can be adjusted through interventions and services that are geared to the unique risk and needs of the individual (Andrews and Bonta 2006; Taxman and Marlowe 2006).

*Health* & *Justice* presents an opportunity, through open access publishing, to advance the agenda to better understand the unique issues associated with individuals involved in the justice system as well as policies, practices, programs, interventions, and services designed to improve health and justice outcomes. The unique challenges of serving the justice-involved population include the degree to which existing policies and practices contribute to health disparities, unequal justice, and further disenhancement from society. The way forward is to reduce the risk to safety and health by new scientific and research endeavors to advance our understanding of policies, clinical practices, and an untold menu of integrated services and systems.

## Authors’ original submitted files for images

Below are the links to the authors’ original submitted files for images. Authors’ original file for figure 1
Authors’ original file for figure 2
