# Supplementary figures and images for: Building effective service delivery mechanisms for justice-involved individuals: an under-researched area
Source: Health Justice. 2014 Jan 29;2:2. doi: 10.1186/2194-7899-2-2 (PMC5151799; doi:10.1186/2194-7899-2-2)

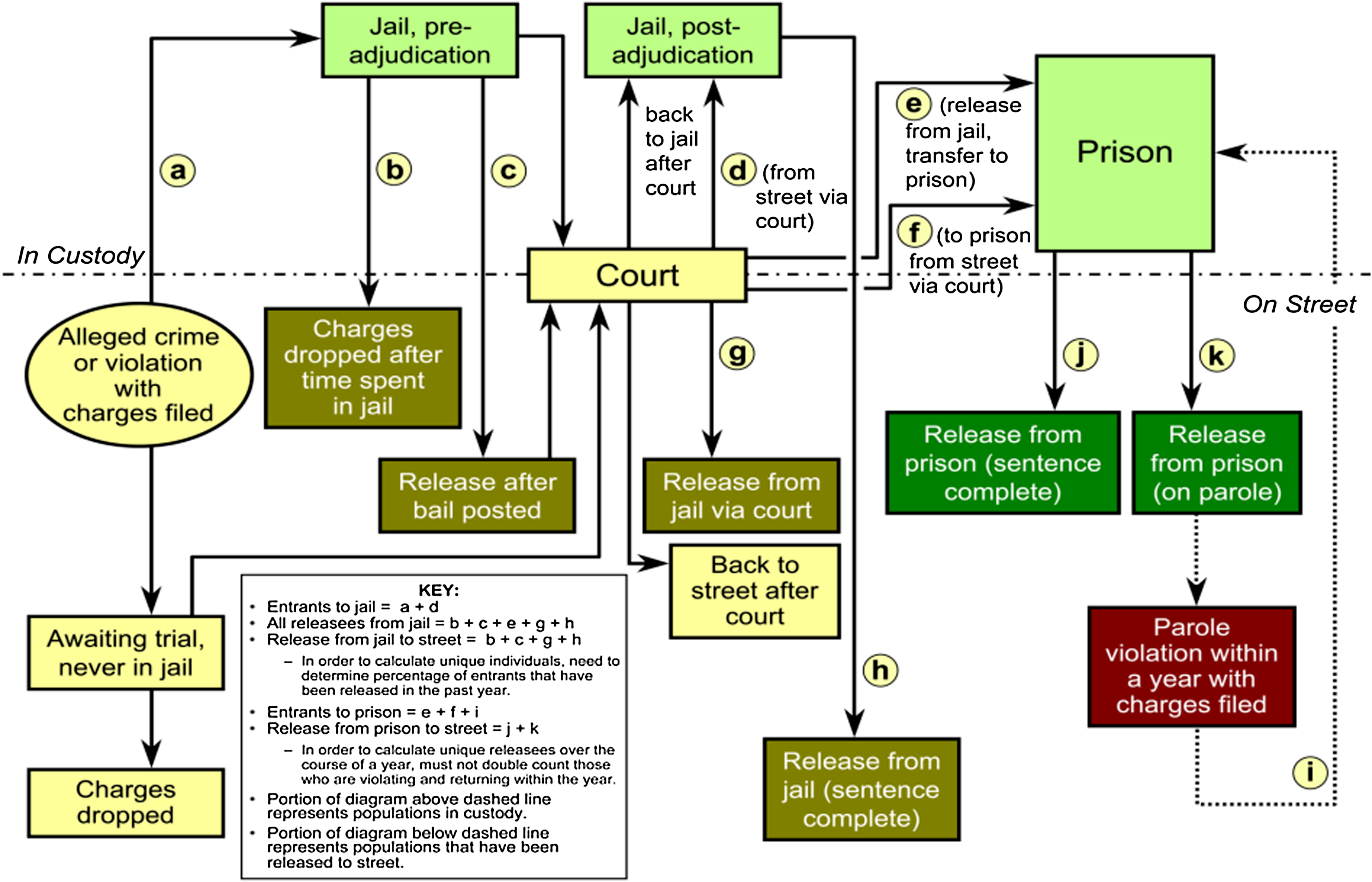

Supplement: Supplementary file 1 — Authors’ original file for figure 1 [file 40352_2013_7_MOESM1_ESM.tiff]

# Eco-Social Model: Need to Address ALL Key Issues

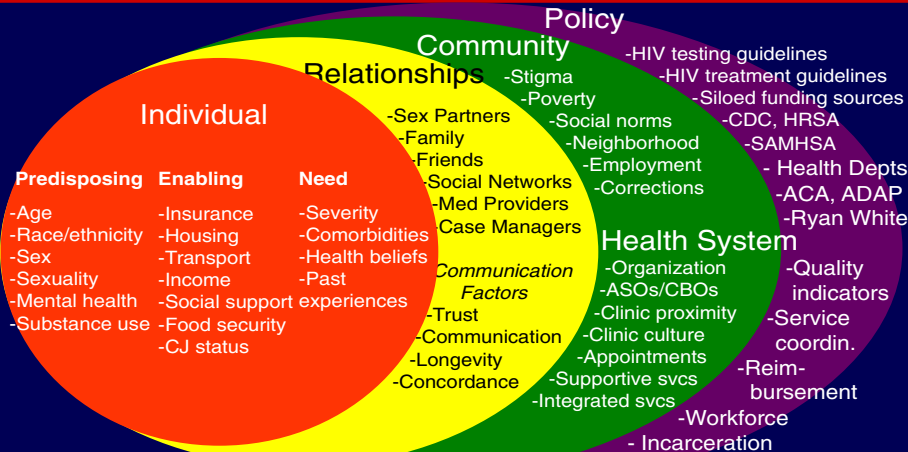

Supplement: Supplementary file 2 — Authors’ original file for figure 2 [file 40352_2013_7_MOESM2_ESM.pdf]
